# Cu_2_O Nanoparticles with Both {100} and {111} Facets for Enhancing the Selectivity and Activity of CO_2_ Electroreduction to Ethylene

**DOI:** 10.1002/advs.201902820

**Published:** 2020-01-30

**Authors:** Yugang Gao, Qian Wu, Xizhuang Liang, Zeyan Wang, Zhaoke Zheng, Peng Wang, Yuanyuan Liu, Ying Dai, Myung‐Hwan Whangbo, Baibiao Huang

**Affiliations:** ^1^ State Key Laboratory of Crystal Materials Shandong University Jinan 250100 China; ^2^ School of Physics Shandong University Jinan 250100 China; ^3^ Department of Chemistry North Carolina State University Raleigh NC 27695‐8204 USA; ^4^ State Key Laboratory of Structural Chemistry Fujian Institute of Research on the Structure of Matter (FJIRSM) Chinese Academy of Sciences (CAS) Fuzhou 350002 China

**Keywords:** crystal facets engineering, cuprous oxide, electrocatalytic CO_2_ reduction, ethylene, selectivity

## Abstract

Cu_2_O nanoparticles (NPs) enclosed with different crystal facets, namely, c‐Cu_2_O NPs with {100} facets, o‐Cu_2_O NPs with {111} facets, and t‐Cu_2_O NPs with both {111} and {100} facets, are prepared and their electrocatalytic properties for the reduction of CO_2_ to C_2_H_4_ are evaluated. It is shown that the selectivity and activity of the C_2_H_4_ production depend strongly on the crystal facets exposed in Cu_2_O NPs. The selectivities for the C_2_H_4_ production increases in the order, c‐Cu_2_O < o‐Cu_2_O < t‐Cu_2_O, (with FE_C2H4_ = 38%, 45%, and 59%, respectively). This study suggests that Cu_2_O NPs are more likely responsible for the selectivity and activity for the C_2_H_4_ production than the metallic Cu NPs produced on the surface of Cu_2_O NPs. This work provides a new route for enhancing the selectivity of the electrocatalytic CO_2_ reduction by crystal facet engineering.

## Graphical abstract

The selectivity of CO_2_ electroreduction to ethylene can be greatly enhanced from Cu_2_O nanoparticles with both {100} and {111} crystal facets exposed, which is ascribed to the better C–C coupling, ethylene desorption and multielectron transfer kinetics. This work provides a new route for enhancing the selectivity of the electrocatalytic reduction of CO_2_ to multicarbon products by crystal facet engineering.

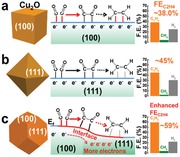

Carbon dioxide (CO_2_) is highly responsible for the global warming and climate change, which makes it urgent to reduce the amount of CO_2_ in atmosphere.^1^, ^2^ In reducing the amount of CO_2_, the electrocatalytic CO_2_ reduction reaction (CO_2_RR) is attractive because it can transform CO_2_ into high‐valued feedstocks with high efficiency and can be combined easily with renewable energy sources such as solar or wind energy.^3^, ^4^, ^5^, ^6^, ^7^ Since the CO_2_RR leads to various carbon products, it is necessary to improve the selectivity and activity for a single desired product. The production of C1 species such as carbon monoxide or formic acid has reached a very high selectivity (over 90%),^8^, ^9^, ^10^, ^11^, ^12^, ^13^, ^14^ but those of multicarbon species with higher commercial values have not.^15^

Among the available electrocatalysts, copper is unique because it can produce various hydrocarbons and alcohols and because it can absorb CO intermediates well, facilitating the subsequent C–C coupling for C_2+_ production.^16^, ^17^, ^18^, ^19^ Ethylene (C_2_H_4_), an important raw material, is one of the main C2 products over Cu electrodes.^20^ However, due to the simultaneous production of H_2_ and other C1 species (i.e., CO, CH_4_), the Faradaic efficiency for the production of C_2_H_4_ (FE_C2H4_) on metallic Cu is usually low.^21^, ^22^, ^23^, ^24^ Efforts to improve the FE_C2H4_ of Cu‐based catalysts have focused on optimizing the sizes, morphologies, and exposed crystal facets of metallic Cu NPs.^25^, ^26^, ^27^, ^28^, ^29^, ^30^, ^31^ In producing C_2_H_4_ with high selectivity, Cu_2_O NPs have recently been found to be more effective than metallic Cu NPs. For example, the FE_C2H4_ of 36% was achieved from a Cu/Cu_2_O catalyst prepared by electro‐redeposition method,^32^ and that of 57% from a nanodendritic Cu catalyst.^33^ Cu_2_O NPs show a good performance probably because the low‐coordinate Cu^+^ ions present on the surface help the C–C coupling, thereby boosting the C_2_H_4_ production.^34^, ^35^, ^36^, ^37^, ^38^, ^39^, ^40^

In controlling the activity and selectivity of electrocatalysts, it is important to understand how they are affected by the crystal facets. The crystal facets of metallic Cu NPs have a strong influence on the selectivity and activity of their catalytic reactions. For example, the Cu {111} facets lead preferentially to CH_4_, while the Cu {100} and some high index planes to C2 products.^33^, ^41^ Studies on Cu_2_O NPs showed that those with different crystal facets exhibit different stabilities and different catalytic activities.^42^, ^43^, ^44^ For example, for the propylene oxidation under high temperature, Cu_2_O NPs enclosed with the {111} facets are more catalytically active than those enclosed with the {100} or {110} facets. During a photocatalytic degradation of methyl orange on Cu_2_O, electron transfer occurs from the {100} and {110} to {111} facets.^45^ During the electrochemical reduction over Cu_2_O under negative potentials, metallic Cu NPs are formed on the surface of Cu_2_O. It has not been unequivocal whether or not active catalysts during CO_2_RR are the metallic Cu NPs produced on the surface of Cu_2_O.^46^, ^47^, ^48^ The Cu NPs produced from Cu_2_O NPs with different morphologies differ in size and aggregation, affecting their selectivity and activity for the C_2_H_4_ production.^39^ These observations prompt us to examine if the metallic Cu NPs derived from Cu_2_O NPs possessing different crystal facets lead to different selectivities and different activities for the C_2_H_4_ production and consequently whether the Cu_2_O or the metallic Cu NPs are responsible for the selectivity and activity of the CO_2_RR.

We explored these questions by preparing Cu_2_O NPs enclosed with different crystal facets, namely, cubic Cu_2_O (c‐Cu_2_O) NPs with {100} facets, octahedral Cu_2_O (o‐Cu_2_O) NPs with {111} facets, and truncated‐octahedral Cu_2_O (t‐Cu_2_O) NPs with both {111} and {100} facets, and then by evaluating the effect of the exposed crystal facets on the selectivity and activity for the C_2_H_4_ production. Our study shows that the selectivity and activity of the C_2_H_4_ production are strongly affected by the crystal facets exposed in Cu_2_O NPs. We show that the selectivities of the Cu_2_O NPs for the C_2_H_4_ production increases in the order, c‐Cu_2_O < o‐Cu_2_O < t‐Cu_2_O, (with FE_C2H4_ = 38%, 45%, and 59%, respectively). Our study suggests strongly that Cu_2_O NPs are more likely responsible for the selectivity and activity for the C_2_H_4_ production than are the metallic Cu NPs produced on the surface of Cu_2_O NPs.

The Cu_2_O NPs were prepared by a wet chemical reduction method.^49^, ^50^ A mixture of Cu_2_O and carbon black was deposited on a glassy carbon electrode (GCE) to form a working electrode for the CO_2_RR (for details, see the Supporting Information). The crystal structure of the samples are determined by X‐ray diffraction (XRD) (Figure S1, Supporting Information). The morphologies of the as‐prepared c‐Cu_2_O, o‐Cu_2_O, and t‐Cu_2_O NPs were characterized by scanning electron microscopy (SEM). The three kinds of Cu_2_O NPs have the sizes of 600–1000 nm, but their morphologies are different. c‐Cu_2_O NPs exhibit cubic nanostructures (**Figure** **1**a,b) enclosed with six {100} planes. The o‐Cu_2_O NPs exhibit an octahedral morphology (Figure 1c,d) exposed with eight Cu_2_O {111} planes. The t‐Cu_2_O NPs exhibit a polyhedral morphology (Figure 1e,f) exposed with both {100} and {111} facets.^45^, ^51^

**Figure 1:**
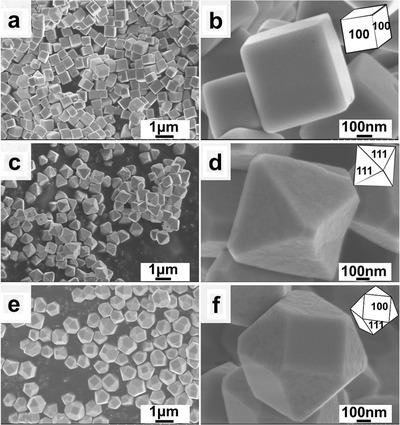
SEM images of a,b) c‐Cu_2_O NPs, c,d) o‐Cu_2_O NPs, and e,f) t‐Cu_2_O NPs.

We now compare the CO_2_RR performances of the three Cu_2_O electrodes by performing potentiostatic measurements in an H‐type electrochemical cell with CO_2_ saturated aqueous 0.5 m KHCO_3_ as electrolyte. The amounts of the gaseous and liquid products were determined by gas chromatography (GC) and nuclear magnetic resonance (NMR), respectively (Figures S2 and S3, Supporting Information). To examine the selectivity for the C_2_H_4_ production, we determine the Faradaic efficiency, FE_C2H4_ = *Q*_C2H4_/*Q*_tot_, where *Q*_C2H4_ is the amount of charge consumed to produce C_2_H_4_, and *Q*_tot_ the charge consumed to produce all products (for details see the Supporting Information). The FE_C2H4_ values for the three Cu_2_O electrodes are compared in **Figure** **2**a. t‐Cu_2_O NPs exhibit the highest selectivity at five selected potentials ranging from −0.9 to −1.3 V relative to reversible hydrogen electrode (RHE) (see Figure S4 in the Supporting Information for RHE calibration). The maximum FE_C2H4_ reaches 59% at −1.1 V, which is comparable to the highest achieved in KHCO_3_ so far by using plasma‐activated copper (60% at −1.1 V).^31^ The maximum FE_C2H4_ for other Cu_2_O NPs are lower, namely, 45% at −1.1 V for o‐Cu_2_O NPs, and 40% at −1.2 V for c‐Cu_2_O NPs. We evaluate the activities of the three Cu_2_O NPs for the C_2_H_4_ production by calculating the currents consumed for the production, *j*_C2H4_ = FE_C2H4_ × *j*_total_, where *j*_total_ is the total current used from the potentiostatic measurements. At any given potential, the *j*_C2H4_ for t‐Cu_2_O NPs is higher than those for o‐Cu_2_O and c‐Cu_2_O NPs (Figure 2b). Furthermore, the *j*_C2H4_ for t‐Cu_2_O and o‐Cu_2_O NPs increases steadily with increasing the potential, but this is not the case for c‐Cu_2_O NPs (Figure 2b). We now examine the stability of Cu_2_O NPs by performing potentiostatic tests at a potential of −1.1 V for 2 h (Figure 2c). The current density of c‐Cu_2_O, o‐Cu_2_O, t‐Cu_2_O NPs is maintained at about 11, 17, and 22 mA cm^−2^, respectively with only ≈5% decrease in 2 h (For more details of the stability test, see Figure S5, Supporting Information). To probe the kinetics of the C_2_H_4_ production, we examine the Tafel plots for the three Cu_2_O NPs (Figure 2d). The Tafel slope for t‐Cu_2_O NPs (75 mV dec^−1^) is lower than those of o‐Cu_2_O (82 mV dec^−1^) and c‐Cu_2_O (97 mV dec^−1^) NPs so that it has the lowest activation energy for the CO_2_RR. In short, for the CO_2_RR toward C_2_H_4_, t‐Cu_2_O NPs exposed with {100} and {111} facets exhibit a better selectivity, activity than do o‐Cu_2_O NPs with {111} facets and c‐Cu_2_O NPs exposed with {100} facets.

**Figure 2:**
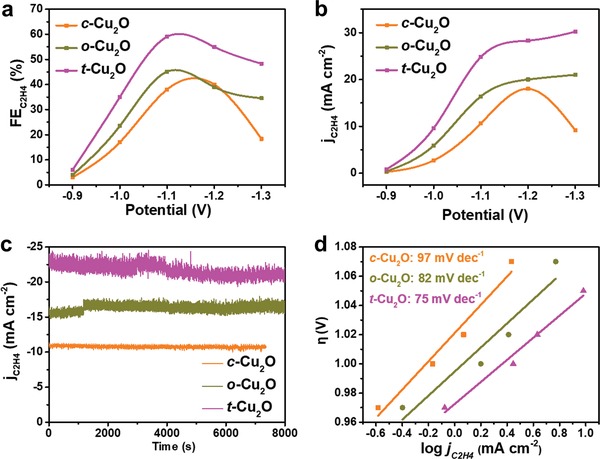
a) FE_C2H4_ values for the c‐Cu_2_O, o‐Cu_2_O, and t‐Cu_2_O NPs as a function of the potential. b) *j*_C2H4_ values for the c‐Cu_2_O, o‐Cu_2_O, and t‐Cu_2_O NPs as a function of the potential. c) *j*_C2H4_ values for the c‐Cu_2_O, o‐Cu_2_O, and t‐Cu_2_O NPs at −1.1 V as a function of the reaction time. d) Tafel plots for the c‐Cu_2_O, o‐Cu_2_O, and t‐Cu_2_O NPs.

In our discussions so far, we have not examined the question whether the catalytic activities of the Cu_2_O NPs are the intrinsic properties of these NPs or they originate from the metallic Cu NPs on the surface of the Cu_2_O NPs produced during the CO_2_RR. To explore this question, we carried out transmission electron microscopy (TEM) measurements for the Cu_2_O NPs after the CO_2_RR. As shown in **Figure** **3**a–c, the morphologies of all the three Cu_2_O nanoparticles can be well preserved after the stability test, with only some tiny nanoparticles on the surfaces, which could be probably ascribed to the Cu nanoparticles formed during electroreduction process. This finding is consistent with the stability tests discussed in Figure 2c.^52^ To further probe the change on composition and valence state of Cu_2_O NPs after electrocatalytic reaction, Cu LMM Auger spectra were performed on the three Cu_2_O samples before and after their use in the CO_2_RR (Figure 3d–f). The peaks of Cu_2_O and Cu are observed at 570.6 and 567.5 eV, respectively. The Auger spectra confirmed that before the stability test, all the three Cu_2_O samples are mainly consisted of Cu^+^. While, a small part (≈5%) of Cu^0^ can be observed beside Cu^+^ after the stability test. According to these results, we conclude that the electrocatalytic activity of Cu_2_O NPs is an intrinsic property of these oxides rather than the metallic Cu NPs produced on their surfaces.

**Figure 3:**
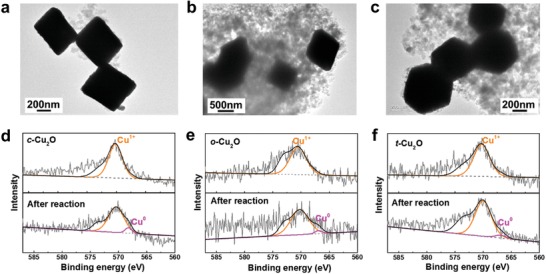
TEM images and Cu LMM Auger spectra of a,d) c‐Cu_2_O, b,e) o‐Cu_2_O, and c,f) t‐Cu_2_O after CO_2_RR, respectively.

To probe a possible reaction mechanism for the CO_2_RR, we examine how the amount of the gaseous products, C_2_H_4_, CH_4_, CO, and H_2_, arising from c‐Cu_2_O, o‐Cu_2_O, and t‐Cu_2_O NPs, vary as a function of the potential as shown in Figure S6 (Supporting Information). The production of C_2_H_4_ and CO over all the three samples exhibit similar trends, where the FE_C2H4_ initially increased sharply then decreased as the applied potential increased, while, the FE_CO_ decreased constantly with the increase of potential. This confirms that adsorbed CO species are intermediates for C_2_H_4_ production during CO_2_RR. Comparing to the production of C_2_H_4_, the formation of CH_4_ is much lower. That indicates C–C coupling to form C_2_H_4_ from adsorbed CO intermediates is preferable than does the formation of CH_4_ on the surface of Cu_2_O NPs. The production of hydrogen from the three Cu_2_O NPs enclosed with different crystal facets are quite different. The FE_H2_ for c‐Cu_2_O is high (≈50%) at −0.9 V versus RHE, which sharply decreased with the increase of applied potential, then increased as the potential is more negative than −1.1 V versus RHE. For o‐Cu_2_O, the FE_H2_ is relatively lower than that from c‐Cu_2_O as the potential is below (≈30%) −1.1 V versus RHE, then increased quickly as the potential further increased. This indicates the production of H_2_ from {100} facets of Cu_2_O could be more preferable than over Cu_2_O {111} facets, which can explain why the FE_C2H4_ over o‐Cu_2_O is higher than that of c‐Cu_2_O. However, for t‐Cu_2_O, the FE_H2_ was kept below 30% as the potential ranging from −0.9 to −1.3 V versus RHE. This indicates the production of H_2_ can be effectively suppressed in t‐Cu_2_O enclosed by both {100} and {111} facets, which lead to the highest FE_C2H4_ among the three samples.

To gain insight into the reason why t‐Cu_2_O has a better performance for the C_2_H_4_ production than does c‐Cu_2_O and o‐Cu_2_O NPs, we carry out DFT calculations to examine the adsorption capabilities of the reaction intermediate CO as well as the product C_2_H_4_ on the Cu_2_O {100} facets, {111} facets and the joint interface between {100} and {111} facets.^53^, ^54^ As shown in **Figure** **4**, CO is more strongly adsorbed on the Cu_2_O {100} facets and the joint interface between {100} and {111} facets than on the Cu_2_O {111} facets. This would subsequently facilitate the C–C coupling to produce C_2+_ products during CO_2_RR. On the other hand, C_2_H_4_ can be adsorbed more weakly on the joint interface between Cu_2_O {100} and {111} facets and Cu_2_O {111} facets than on the Cu_2_O {100} facets. That means c‐Cu_2_O enclosed by {100} facets could facilitate the C–C coupling to produce C_2+_ products, but the formed C_2+_ products (e.g., C_2_H_4_) can hardly escape from the surface of Cu_2_O {100} facet because of its stronger adsorption ability of C_2_H_4_. For o‐Cu_2_O enclosed by {111} facets, although the adsorption of CO intermediates is lower than that on Cu_2_O {100} facets, once C_2_H_4_ was formed, it can be easily desorbed from the surface of Cu_2_O {111} facets because of its weaker adsorption ability. Meanwhile, for t‐Cu_2_O enclosed by both {100} and {111} facets, the CO intermediates can not only be strongly adsorbed on the joint interface between {100} and {111} facets to promote the C–C coupling, but also the as‐formed C_2_H_4_ can be easily desorbed from the joint interface to promote the C_2_H_4_ production.

**Figure 4:**
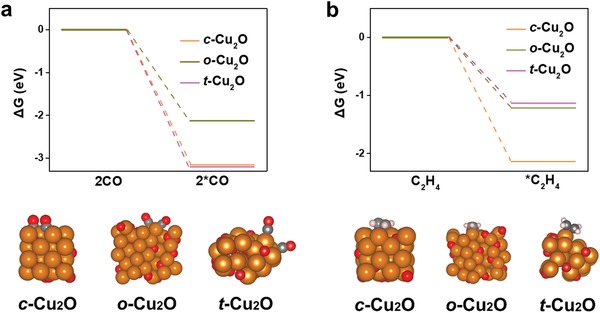
Adsorption energies of a) CO and b) C_2_H_4_ on the {100} surfaces, {111} surfaces and the interface of {100} and {111} surfaces of Cu_2_O. The corresponding adsorption configurations are also shown.

In addition, the reason why t‐Cu_2_O provides a much better catalytic performance than does c‐Cu_2_O and o‐Cu_2_O may be related to the fact that the Fermi level of Cu_2_O is lower on the {111} than on the {100} facets.^54^ This could subsequently facilitate the charge transfer between Cu_2_O {111} and {100} facets, and further promote the multielectron involved kinetics for ethylene production in Cu_2_O nanoparticles enclosed by both {111} and {100} facets (**Figure** **5**a–c).^55^, ^56^, ^57^

**Figure 5:**
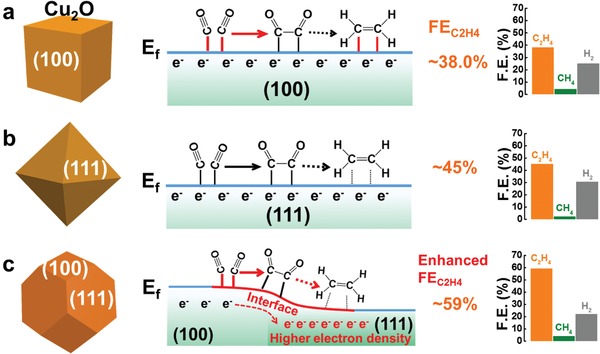
Formation of C_2_H_4_ on the a) {100} facets of c‐Cu_2_O NPs, b) {111} facets of o‐Cu_2_O NPs, and c) {100} and {111} facets of t‐Cu_2_O NPs.

In summary, the t‐Cu_2_O NPs enclosed with both {100} and {111} facets exhibit the FE_C2H4_ and *j*_C2H4_ values of 59% and 23.1 mA cm^−2^, respectively, for the CO_2_RR at −1.1 V in 0.5 m KHCO_3_. These are better than those of the o‐Cu_2_O NPs with {111} facets (45%, 16.4 mA cm^−2^) and c‐Cu_2_O NPs with {100} facets (38%, 10.6 mA cm^−2^). Our study suggests that the electrocatalytic activity of Cu_2_O NPs is an intrinsic property of these oxides rather than the metallic Cu NPs produced on their surfaces during the CO_2_RR. The enhanced performance of t‐Cu_2_O NPs can be attributed to the synergistic effect of {100} and {111} facets, which can not only facilitate the C–C coupling and C_2_H_4_ desorption, but also be able to promote the multielectron involved kinetics for ethylene production. Our work may provide a new route for enhancing the selectivity of the electrocatalytic CO_2_ reduction by crystal facet engineering.

## Experimental Section

##### Preparation of the c‐Cu_2_O, o‐Cu_2_O, and t‐Cu_2_O Particles

Cu_2_O particles were synthesized by wet chemical reduction method according to previous reports.^1^, ^2^ In a typical synthesis, polyvinylpyrrolidone (PVP, MW 24 000) (0 g for c‐Cu_2_O, 4 g for t‐Cu_2_O, and 6 g for o‐Cu_2_O) was added into 100 mL CuCl_2_ · 2H_2_O aqueous solution. Then, 10.0 mL NaOH aqueous solution (2.0 m) was added dropwise into the above solution. After stirring for 30 min, 10.0 mL ascorbic acid solution (0.60 m) was added dropwise into the dark brown solution. The mixture was aged for 3 h and the solution gradually transferred into turbid red. All of the procedure was carried out under constant stirring and heated in a water bath at 55 °C. The resulting precipitate was collected by centrifugation and decanting, followed by washing with distilled water 3 times and absolute ethanol 3 times and finally dried under vacuum at 60 °C for 6 h.

##### Preparation of the Electrodes

Typically, an ink of Cu_2_O/C particles was prepared by adding 2 mg catalyst (c‐Cu_2_O, o‐Cu_2_O, or t‐Cu_2_O particles) and 8 mg Carbon Black into the ink‐base of 800 µL of isopropanol, 100 µL of H_2_O, and 100 µL of 5% nafion solution and then ultrasonicating the solution for 3 h. We deposited 10 µL of the sample inks on the GCE (diameter, 5 mm) to form the sample electrodes.

##### Electrochemical Experiments

The linear sweeping voltammetry (LSV) measurements were carried out with an Ag/AgCl reference electrode (with saturated KCl as the filling solution), a platinum electrode as the counter electrode and the as‐prepared samples as the working electrode. The product analysis was carried out in a two‐compartment electrochemical cell with an anion exchange membrane separating the working and counter electrodes. The potentiostatic measurements were performed using a three‐electrode system to determine the value of the consumed coulomb, and the amounts of the gases produced were measured by the GC and GCMS instruments. The electrolyte was potassium bicarbonate saturated with CO_2_ by bubbling high‐purity CO_2_ gas, before each experiment, at a flow rate of 50 mL min^−1^ for 1 h to remove all oxygen from the electrolyte. The working electrode was tested 20 times before the plot is recorded at a scan rate of 50 mV s^−1^. All potentials were transformed to the reversible hydrogen electrode reference by using the calibrated relationship, *E*_RHE_ = *E*_Ag/AgCl_ + 0.657 V.

##### Characterization

Crystal structures of the as‐obtained products were characterized by XRD measurements with a Bruker AXS D8 diffractometer using Cu Kα radiation. Fourier transform infrared (FTIR) spectra were obtained on a Bruker ALPHA‐T spectrometer using KBr pellets. Raman spectra were recorded on a microscopic confocal Raman spectrometer (Horiba JobinYvon, LabRAM HR) with an excitation of 613 nm laser light. Morphologies and microstructures of the products were characterized by scanning electron microscopy (Hitachi S‐4800) equipped with an Energy Dispersive Spectrometer (EDS) and transmission electron microscopy using a Philips Tecnai 20U‐Twin microscope at an acceleration voltage of 200 kV. (JEOL JEM‐2100F). X‐ray photoelectron spectroscopy (XPS) measurement was performed using a Thermo Fisher Scientific Escalab 250 spectrometer with monochromatized Al Kα excitation, and C1s (284.6 eV) was used to calibrate the peak positions of various elements. All electrochemical experiments were carried out using the electrochemical workstation CHI660E. The gas products from the compartment were examined with a gas chromatograph equipped with a TDX‐01 column with a flame ionization detector (FID) and a H_2_‐detection GC (ShiweipxGC‐7806) with a thermal conductivity detector (TCD). Gas chromatograph‐mass spectrometer (GCMS) was used to determine the concentration of liquid products with a Max capillary column.

The faradaic efficiency (FE) was calculated by the following equation

(1) $$FE_{\text{C}2\text{H}4}=\frac{\alpha nF}{Q}\;=\;\frac{2nF}{It}$$

Where α is the number of the electrons transferred for CO, *F* is the Faraday constant, *Q* is the charge, *I* is the current, *t* is the running time, and *n* is the total amount of CO (in moles).

## Conflict of Interest

The authors declare no conflict of interest.

## Supporting information

Supporting Information

## References

[1] B. Obama , Science 2017, 355, 126. 28069665 10.1126/science.aam6284

[2] M. Mikkelsen , M. Jørgensen , F. C. Krebs , Energy Environ. Sci. 2010, 3, 43.

[3] H. Q Yang , Z. H. Xu , M. H. Fan , R. Gupta , R. B. Slimane , A. E. Bland , I. Wright , J. Environ. Sci. 2008, 20, 14. 10.1016/s1001-0742(08)60002-9 18572517

[4] J. Qiao , Y. Liu , F. Hong , J. Zhang , Chem. Soc. Rev. 2014, 43, 631. 24186433 10.1039/c3cs60323g

[5] L. Zhang , Z. J. Zhao , J. Gong , Angew. Chem., Int. Ed. 2017, 56, 11326. 10.1002/anie.201612214 28168799

[6] S. Chu , Y. Cui , N. Liu , Nat. Mater. 2017, 16, 16. 10.1038/nmat4834 27994253

[7] C. Liu , B. C. Colon , M. Ziesack , P. A. Silver , D. G. Nocera , Science 2016, 352, 1210. 27257255 10.1126/science.aaf5039

[8] S. Gao , Y. Lin , X. Jiao , Y. Sun , Q. Luo , W. Zhang , D. Li , J. Yang , Y. Xie , Nature 2016, 529, 68. 26738592 10.1038/nature16455

[9] S. Liu , H. Tao , L. Zeng , Q. Liu , Z. Xu , Q. Liu , J. L. Luo , J. Am. Chem. Soc. 2017, 139, 2160. 28150946 10.1021/jacs.6b12103

[10] Y. Gao , F. Li , P. Zhou , Z. Wang , Z. Zheng , P. Wang , Y. Liu , Y. Dai , M. Whangbo , B. Huang , J. Mater. Chem. A 2019,7, 16685.

[11] Q. Lu , J. Rosen , Y. Zhou , G. S. Hutchings , Y. C. Kimmel , J. G. Chen , F. Jiao , Nat. Commun. 2014, 5, 3242. 24476921 10.1038/ncomms4242

[12] B. A. Rosen , A. Salehi‐Khojin , M. R. Thorson , W. Zhu , D. T. Whipple , P J. A. Kenis , R. I. Masel , Science 2011, 334, 643. 21960532 10.1126/science.1209786

[13] M. Liu , Y. Pang , B. Zhang , P. De Luna , O. Voznyy , J. Xu , X. Zheng , C. T. Dinh , F. Fan , C. Cao , F. P. de Arquer , T. S. Safaei , A. Mepham , A. Klinkova , E. Kumacheva , T. Filleter , D. Sinton , S. O. Kelley , E. H. Sargent , Nature 2016, 537, 382. 27487220 10.1038/nature19060

[14] Q. Gong , P. Ding , M. Xu , X. Zhu , M. Wang , J. Deng , Q. Ma , N. Han , Y. Zhu , J. Lu , Z. Feng , Y. Li , W. Zhou , Y. Li , Nat. Commun. 2019, 10, 2807. 31243275 10.1038/s41467-019-10819-4 PMC6594929

[15] P. D. Luna , C. Hahn , D. Higgins , S. A. Jaffer , T. F. Jaramillo , E. H. Sargent , Science 2019, 364, eaav3506. 31023896 10.1126/science.aav3506

[16] Y. Hori , Electrochemical CO_2_ Reduction on Metal Electrodes, Modern Aspects of Electrochemistry, Vol. 42 (Eds: Vayenas C. G., White R. E., Gamboa‐Aldeco M. E.), Springer, New York, NY 2008.

[17] K. Ogura , H. Yano , F. Shirai , J. Electrochem. Soc. 2003, 150, D163.

[18] A. A. Peterson , F. Abild‐Pedersen , F. Studt , J. Rossmeisl , J. K. Nørskov , Energy Environ. Sci. 2010, 3, 1311.

[19] K. P. Kuhl , E. R. Cave , D. N. Abram , T. F. Jaramillo , Energy Environ. Sci. 2012, 5, 7050.

[20] J. Hussain , H. Jónsson , E. Skúlason , ACS Catal. 2018, 8, 5240.

[21] J. Wang , F. Zhang , X. Kang , S. Chen , Curr. Opin. Electrochem. 2019, 13, 40.

[22] O. A. Baturina , Q. Lu , M. A. Padilla , L. Xin , W. Li , A. Serov , K. Artyushkova , P. Atanassov , F. Xu , A. Epshteyn , T. Brintlinger , M. Schuette , G. E. Collins , ACS Catal. 2014, 4, 3682.

[23] Y. X. Duan , F. L. Meng , K. H. Liu , S. S. Yi , S. J. Li , J. M. Yan , Q. Jiang , Adv. Mater. 2018, 30, 1706194. 10.1002/adma.201706194 29473227

[24] Y. Wang , Z. Chen , P. Han , Y. Du , Z. Gu , X. Xu , G. Zheng , ACS Catal. 2018, 8, 7113.

[25] K. J. Schouten , Z. Qin , E. Perez Gallent , M. T. Koper , J. Am. Chem. Soc. 2012, 134, 9864. 22670713 10.1021/ja302668n

[26] Y. Hori , I. Takahashi , O. Koga , N. Hoshi , J. Phys. Chem. B 2002, 106, 15.

[27] X. Liu , J. Xiao , H. Peng , X. Hong , K. Chan , J. K. Norskov , Nat. Commun. 2017, 8, 15438. 28530224 10.1038/ncomms15438 PMC5458145

[28] A. Verdaguer‐Casadevall , C. W. Li , T. P. Johansson , S. B. Scott , J. T. McKeown , M. Kumar , I. E. Stephens , M. W. Kanan , I. Chorkendorff , J. Am. Chem. Soc. 2015, 137, 9808. 26196863 10.1021/jacs.5b06227

[29] C. W. Li , J. Ciston , M. W. Kanan , Nature 2014, 508, 504. 24717429 10.1038/nature13249

[30] D. Gao , I. Zegkinoglou , N. J. Divins , F. Scholten , I. Sinev , P. Grosse , B. Roldan Cuenya , ACS Nano 2017, 11, 4825. 28441005 10.1021/acsnano.7b01257

[31] H. Mistry , A. S. Varela , C. S. Bonifacio , I. Zegkinoglou , I. Sinev , Y. W. Choi , K. Kisslinger , E. A. Stach , J. C. Yang , P. Strasser , B. R. Cuenya , Nat. Commun. 2016, 7, 12123. 27356485 10.1038/ncomms12123 PMC4931497

[32] P. D. Luna , R. Quintero‐Bermudez , C. Dinh , M. B. Ross , O. S. Bushuyev , P. Todorović , T. Regier , S. O. Kelley , P. Yang , E. H. Sargent , Nat. Catal. 2018, 1, 103.

[33] C. Reller , R. Krause , E. Volkova , B. Schmid , S. Neubauer , A. Rucki , M. Schuster , G. Schmid , Adv. Energy Mater. 2017, 7, 1602114.

[34] Y. Lum , J. W. Ager , Angew. Chem., Int. Ed. 2018, 57, 551. 10.1002/anie.201710590 29110417

[35] Z. Q. Liang , T. T. Zhuang , A. Seifitokaldani , J. Li , C. W. Huang , C. S. Tan , Y. Li , P. De Luna , C. T. Dinh , Y. Hu , Q. Xiao , P. L. Hsieh , Y. Wang , F. Li , R. Quintero‐Bermudez , Y. Zhou , P. Chen , Y. Pang , S. C. Lo , L. J. Chen , H. Tan , Z. Xu , S. Zhao , D. Sinton , E. H. Sargent , Nat. Commun. 2018, 9, 3828. 30237471 10.1038/s41467-018-06311-0 PMC6148248

[36] M. Favaro , H. Xiao , T. Cheng , W. A. Goddard III , J. Yanoa , E. J. Crumlin , Proc. Natl. Acad. Sci. USA 2017, 114, 6706. 28607092 10.1073/pnas.1701405114 PMC5495248

[37] H. Xiao , W. A. Goddard III , T. Cheng , Y. Liu , Proc. Natl. Acad. Sci. USA 2017, 114, E7045.28784782

[38] H. Jung , S. Y. Lee , C. W. Lee , M. K. Cho , D. H. Won , C. Kim , H. S. Oh , B. K. Min , Y. J. Hwang , J. Am. Chem. Soc. 2019, 141, 4624. 30702874 10.1021/jacs.8b11237

[39] C. W. Li , M. W. Kanan , J. Am. Chem. Soc. 2012, 134, 7231. 22506621 10.1021/ja3010978

[40] A. Eilert , F. Cavalca , F. S. Roberts , J. Osterwalder , C. Liu , M. Favaro , E. J. Crumlin , H. Ogasawara , D. Friebel , L. G. M. Pettersson , A. Nilsson , J. Phys. Chem. Lett. 2017, 8, 285. 27983864 10.1021/acs.jpclett.6b02273

[41] Y. Hori , I. Takahashi , O. Koga , N. Hoshi , J. Mol. Catal. A: Chem. 2003, 199, 39.

[42] W. Huang , Acc. Chem. Res. 2016, 49, 520. 26938790 10.1021/acs.accounts.5b00537

[43] K. Jiang , R. B. Sandberg , A. J. Akey , X. Liu , D. C. Bell , J. K. Nørskov , K. Chan , H. Wang , Nat. Catal. 2018, 1, 111.

[44] X. Qin , P. B. Balbuena , M. Shao , J. Phys. Chem. C 2019, 123, 14449.

[45] Z. Zheng , B. Huang , Z. Wang , M. Guo , X. Qin , X. Zhang , P. Wang , Y. Dai , J. Phys. Chem. C 2009, 113, 14448.

[46] Z. Wang , G. Yang , Z. Zhang , M. Jin , Y. Yin , ACS Nano 2016, 10, 4559. 26974506 10.1021/acsnano.6b00602

[47] S. Lee , D. Kim , J. Lee , Angew. Chem., Int. Ed. 2015, 54, 14701. 10.1002/anie.201505730 26473324

[48] D. Ren , Y. Deng , A. D. Handoko , C. S. Chen , S. Malkhandi , B. S. Yeo , ACS Catal. 2015, 5, 2814.

[49] D.‐F. Zhang , H. Zhang , L. Guo , K. Zheng , X.‐D. Han , Z. Zhang , J. Mater. Chem. 2009, 19, 5220.

[50] J. Lin , W. Hao , Y. Shang , X. Wang , D. Qiu , G. Ma , C. Chen , S. Li , L. Guo , Small 2018, 14, 1703274. 10.1002/smll.201703274 29239098

[51] Y. Sui , W. Fu , Y. Zeng , H. Yang , Y. Zhang , H. Chen , Y. Li , M. Li , G. Zou , Angew. Chem., Int. Ed. 2010, 49, 4282. 10.1002/anie.200907117 20446323

[52] P. Zhou , D. Xing , Y. Liu , Z. Wang , P. Wang , Z. Zheng , X. Qin , X. Zhang , Y. Dai , B. Huang , J. Mater. Chem. A 2019, 7, 5513.

[53] S. Back , M. S. Yeom , Y. Jung , ACS Catal. 2015, 5, 5089.

[54] H. L. Skriver , N. M. Rosengaard , Phys. Rev. B 1992, 46, 7157. 10.1103/physrevb.46.7157 10002423

[55] N. Shehzad , M. Tahir , K. Johari , T. Murugesan , M. Hussain , J. CO2 Util. 2018, 26, 98.

[56] Z. Xiong , Z. Lei , C.‐C. Kuang , X. Chen , B. Gong , Y. Zhao , J. Zhang , C. Zheng , J. C. S. Wu , Appl. Catal., B 2017, 202, 695.

[57] S. Xie , Y. Wang , Q. Zhang , W. Deng , Y. Wang , ACS Catal. 2014, 4, 3644.

