# Supplementary material for: Cu2O Nanoparticles with Both {100} and {111} Facets for Enhancing the Selectivity and Activity of CO2 Electroreduction to Ethylene
Source: Adv Sci (Weinh). 2020 Jan 30;7(6):1902820. doi: 10.1002/advs.201902820 (PMC7080533; doi:10.1002/advs.201902820)
Supplement: Supplementary file 1 — Supporting Information [file ADVS-7-1902820-s001.pdf]

Copyright WILEY-VCH Verlag GmbH & Co. KGaA, 69469 Weinheim, Germany, 2020.

ADVANCED SCIENCE
Open Access

Supporting Information

for *Adv. Sci.,* DOI: 10.1002/advs.201902820

Cu_2O Nanoparticles with Both {100} and {111} Facets for Enhancing the Selectivity and Activity of CO_2 Electroreduction to Ethylene

Yugang Gao, Qian Wu, Xizhuang Liang, Zeyan Wang, Zhaoke Zheng, Peng Wang, Yuanyuan Liu, Ying Dai, Myung-Hwan Whangbo, and Baibiao Huang*

Supplementary Information

Cu_2O particles with both {100} and {111} facets for enhancing the selectivity and activity of CO_2 electroreduction to ethylene

Yugang Gao[a], *Qian Wu*[b], *Xizhuang Liang*[a], *Zeyan Wang*[a,]*, *Zhaoke Zheng*[a], *Peng Wang*[a], *Yuanyuan Liu*[a], *Ying Dai*[b], *Myung-Hwan Whangbo*[a,c,d], *Baibiao Huang*[a,]*

[a] State Key Laboratory of Crystal Materials, Shandong University, Jinan 250100, China

[b] School of Physics, Shandong University, Jinan 250100, China

[c] Department of Chemistry, North Carolina State University, Raleigh, North Carolina 27695-8204, USA

[d] State Key Laboratory of Structural Chemistry, Fujian Institute of Research on the Structure of Matter (FJIRSM), Chinese Academy of Sciences (CAS), Fuzhou 350002, China

Corresponding authors:

Email: wangzeyan@sdu.edu.cn, bbhuang@sdu.edu.cn

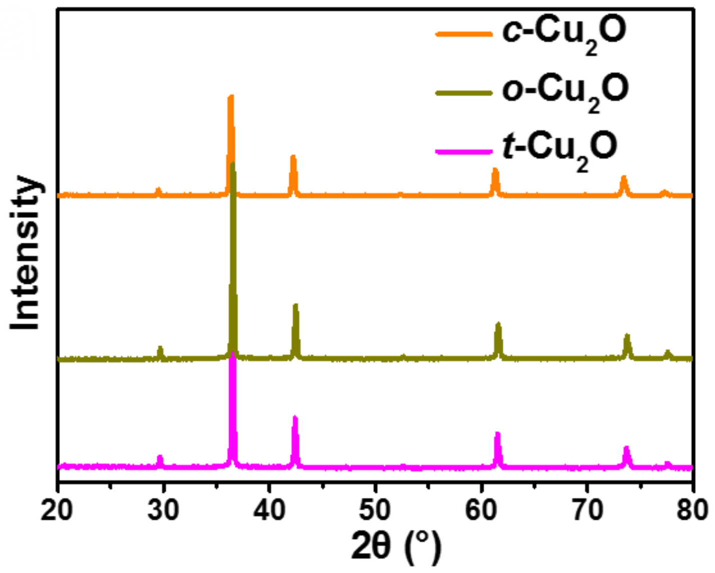

Figure S1. XRD patterns for *c*-Cu_2O, *o*-Cu_2O and *t*-Cu_2O particles.

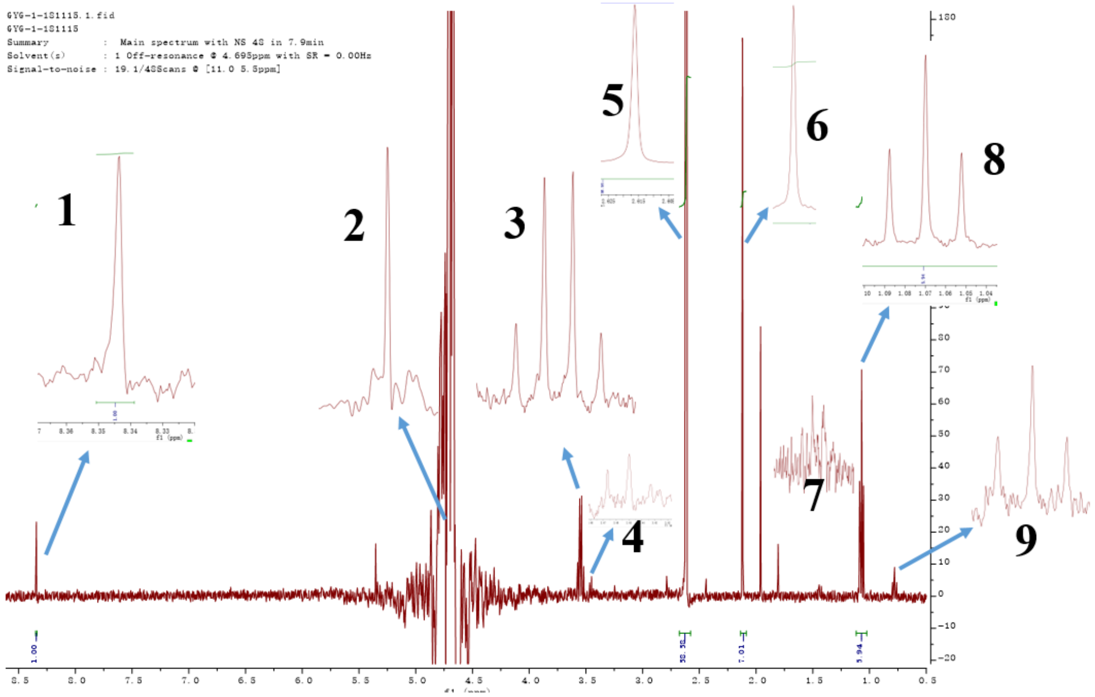

Figure S2. NMR spectrum of the liquid products for the *t*-Cu_2O electrode.

1 HCOOH 2 H_2O 3,8 ethanol 5 DMSO 6 CH_3COOH 4, 7 ,9 $CH_3CH_2CH_2OH$

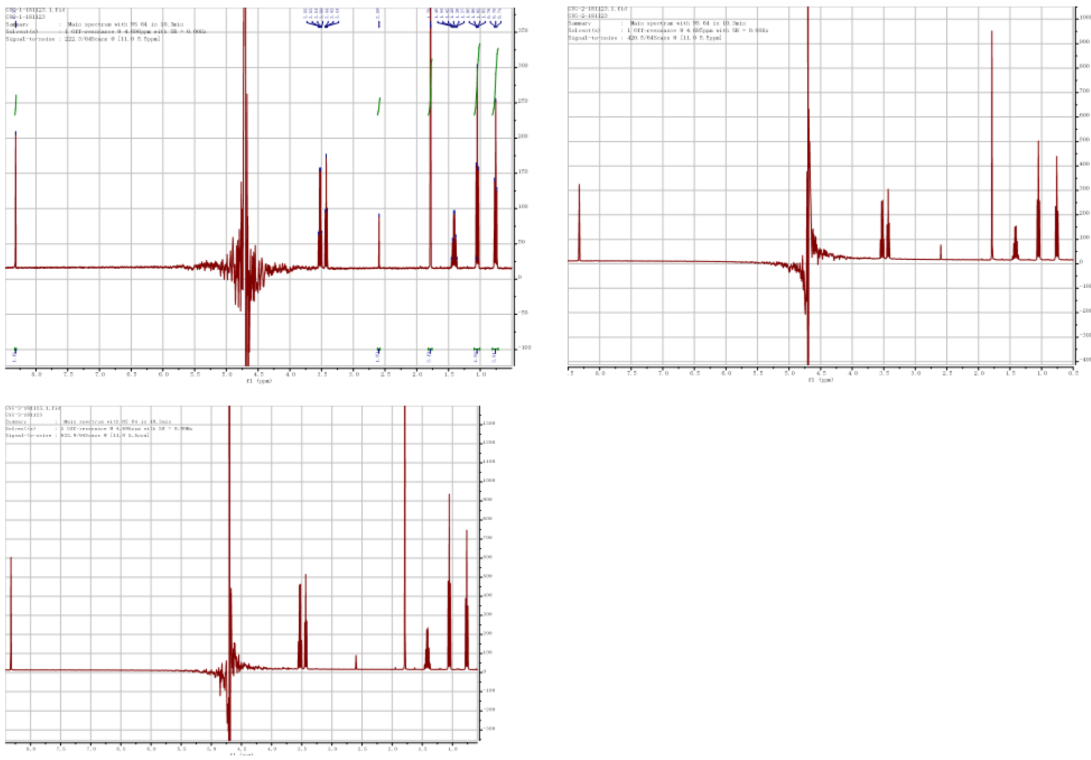

Figure S3. Calibration of the liquid products by using n-propanol, ethanol, formic acid and acetic acid.

RHE calibration

The RHE calibration is performed under the practical condition according to the previous reports (**Figure S4**) [3,4]. The calibration was carried out in the high purity hydrogen and CO_2 saturated $KHCO_3$ electrolyte with a Pt wire as the working electrode. Cycle Voltammetry was run at a scan rate of 1 mV s^{-1}. The average of the two potentials at which the current crossed zero was taken to be the thermodynamic potential for the hydrogen electrode reactions. So in CO_2 saturated 0.5M $KHCO_3$, E (RHE) = E (Ag/AgCl) + 0.657 V.

CO_2 saturated 0.5M $KHCO_3$

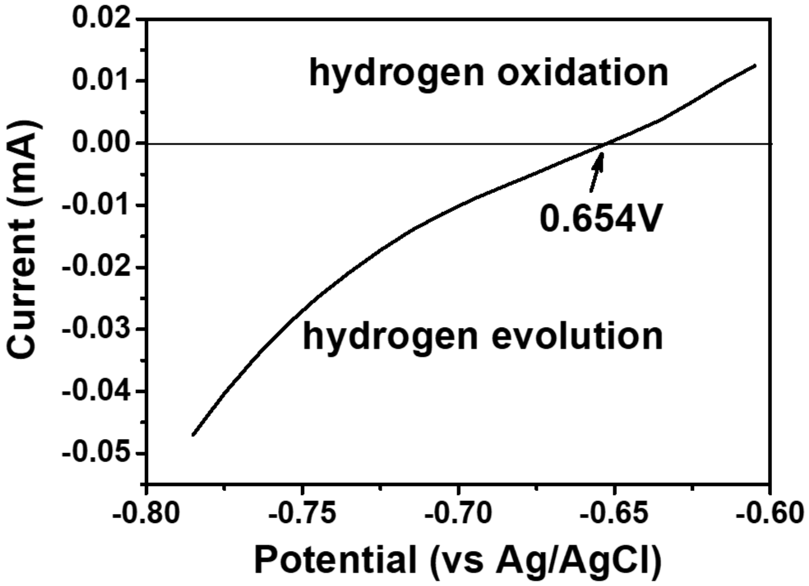

Figure S4. Calibration of Ag/AgCl reference electrode relative to the RHE.

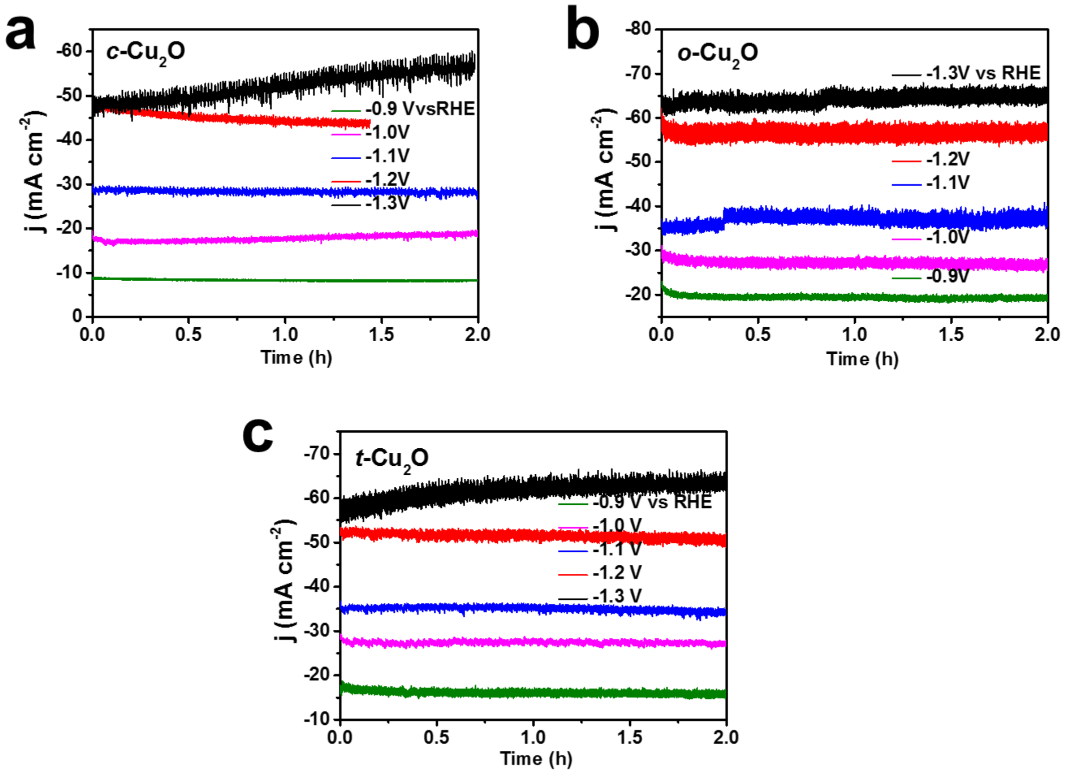

Figure S5. Stability tests for (a) *c*-Cu_2O, (b) *o*-Cu_2O and (c) *t*-Cu_2O electrodes at various potentials.

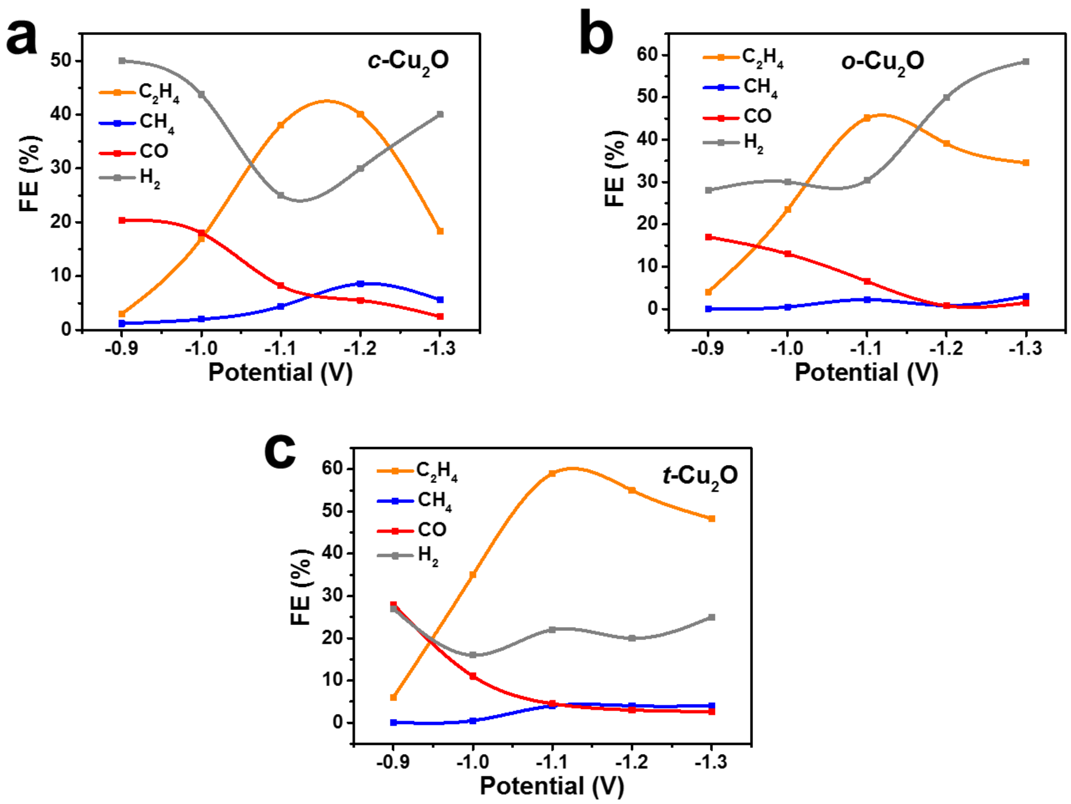

Figure S6. Selectivity FE for the C_2H_4, CH_4, CO and H_2 production of (a) the *c*-Cu_2O electrode, (b) the *o*-Cu_2O electrode, (c) the *t*-Cu_2O electrode at -0.9~-1.3V vs RHE.

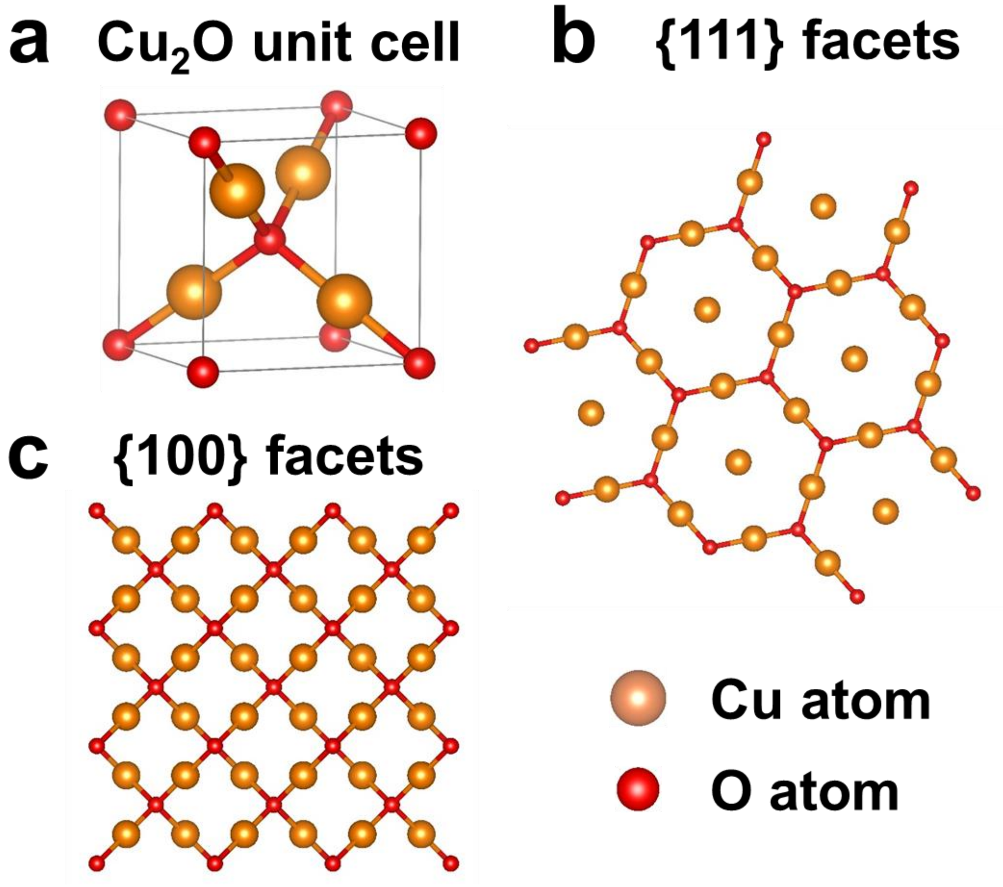

Figure S7. Illustration of the a) unit cell of cuprite Cu_2O, and b, c) the crystal structure of Cu_2O {111} and {100} facets, respectively.

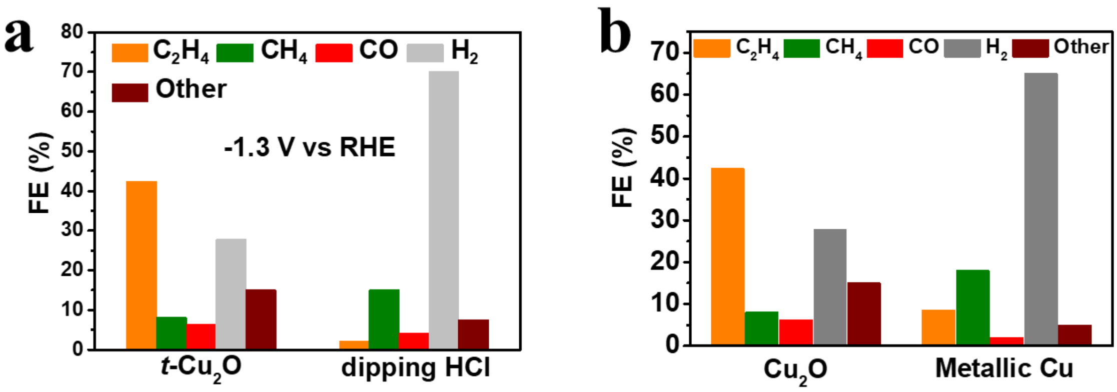

Figure S8. (a) Faradic efficiency for the CO_2RR of the *t*-Cu_2O electrode before and after dipping HCl solution (b) Faradic efficiency of the *t*-Cu_2O electrode and metallic Cu electrode.

Computational section

All calculations were performed by using the Vienna Ab Initio Simulation Package (VASP)[5,6] with the projector augmented wave (PAW) method.[6] We employed the generalized gradient approximation (GGA) of the Perdew−Burke−Ernzerhof (PBE) exchange-correlation functional[7] and a cutoff energy of 500 eV. The van der Waals (vdW) interactions were taken into account by the empirical correction scheme of Grimme (DFT+D2).[8] A Monkhorst-Pack 5 × 5 × 1 k-point grid was adopted for all the calculations. The convergence criterion for the residual force and energy was set to 0.01 eV/Å and 10^{-4} eV with a vacuum space larger than 20 Å in the z direction to avoid interactions between periodic units during the structure relaxation.

The computational hydrogen electrode (CHE)[9] model was used to compute the Gibbs reaction free energy change (ΔG) of each elementary step for NOER. The ΔG of every step is obtained by

$$\Delta G = \Delta E + \Delta E_{\mathrm{ZPE}} - T\Delta S$$

where ΔE is the enthalpy difference from DFT computations, ΔE_{ZPE} is the change in zero point energy, T and ΔS is the temperature (298.15 K) and the entropy changes, which can be calculated from the vibrational frequencies.

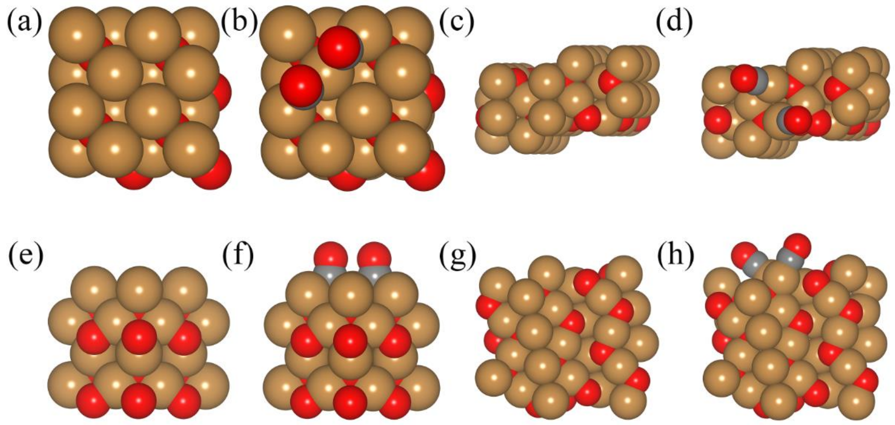

Figure S9. (a, b) Top views of the (100) facets before and after the CO adsorption. (c, d) Top views of the (111) facets before and after the CO adsorption. (e, f) Side views of the (100) facets before and after CO adsorption. (g, h) Side views of the (111) facets before and after the CO adsorption.

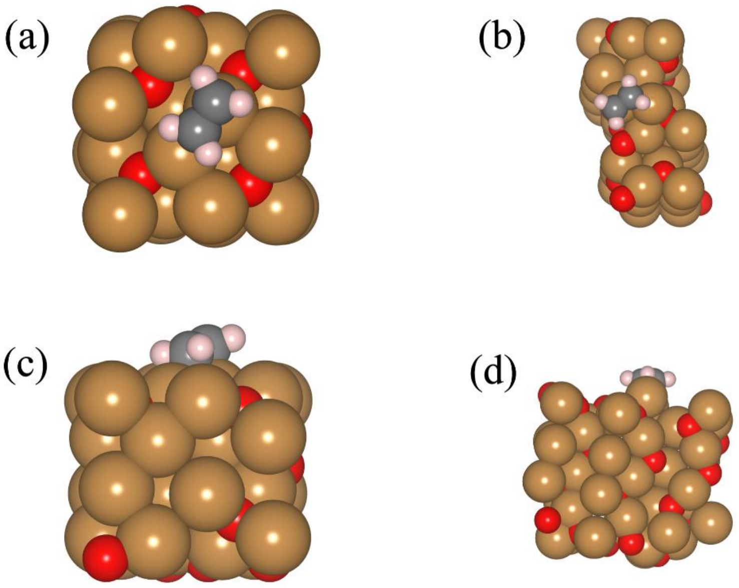

Figure S10. (a, b) Top views of the (100) and (111) facets after the ethylene adsorption. (c, d) Side views of the (100) and (111) facets after the ethylene adsorption.

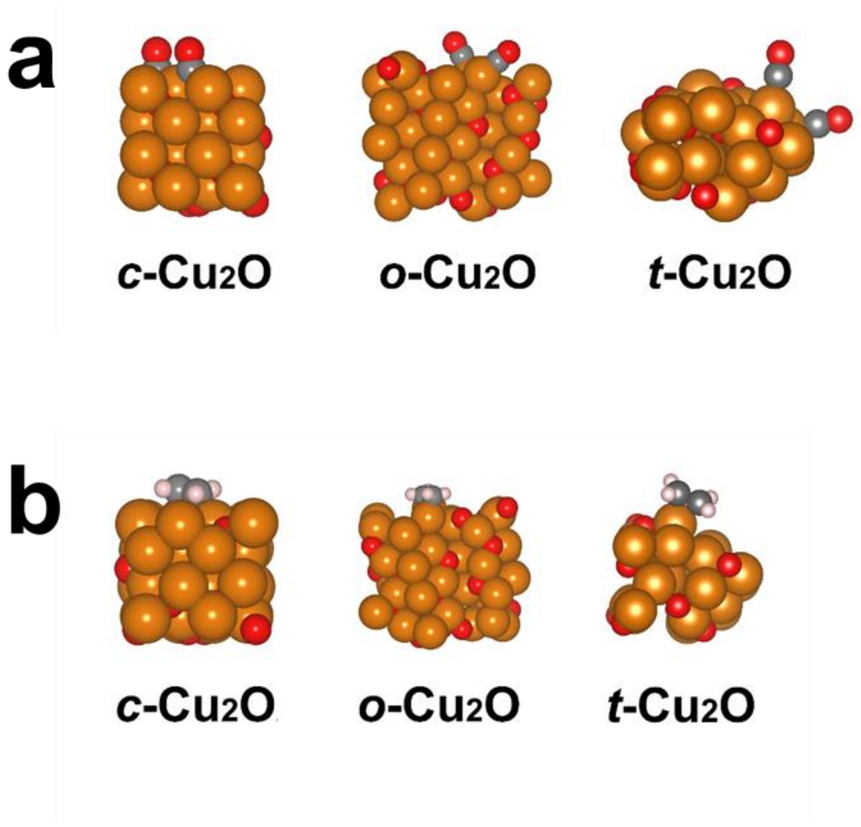

Figure S11. Structure of the {100}, {111} facets and the interface between this two facets after (a) CO adsorption. (b) Ethylene adsorption.

References

[1] D.-F. Zhang, H. Zhang, L. Guo, K. Zheng, X.-D. Han, Z. Zhang, *Journal of Materials Chemistry* **2009**, 19, 5220.

[2] J. Lin, W. Hao, Y. Shang, X. Wang, D. Qiu, G. Ma, C. Chen, S. Li, L. Guo, *Small* **2018**, 14, 1703274.

[3] Y. Liang, Y. Li, H. Wang, J. Zhou, J. Wang, T. Regier and H. Dai, Nat Mater, **2011**, 10, 780-786.

[4] Y. Li, W. Zhou, H. Wang, L. Xie, Y. Liang, F. Wei, J. C. Idrobo, S. J. Pennycook and H. Dai, Nat Nanotechnol, **2012**, 7, 394-400.

[5] G. Kresse, J. Furthmüller, J. *Phys. Rev. B: Condens. Matter Mater. Phys*. **1996**, *54*, 11169.

[6] G. Kresse, D.Joubert, *Phys. Rev. B: Condens. Matter Mater. Phys*. **1999**, *59*, 1758−1775.

[7] J. P. Perdew, K. Burke, Ernzerhof, M. *Phys. Rev. Lett.* **1996**, *77*, 3865.

[8] S. Grimme, *J. Comput. Chem.*, **2006**, *27*, 1787–1799.

[9] J. K. Nørskov, J. Rossmeisl, A. Logadottir, L. Lindqvist, J. R. Kitchin, T. Bligaard, H. J´onsson, *J. Phys. Chem. B* **2004**, *108*, 17886–17892.
